# ML264 inhibits osteosarcoma growth and metastasis via inhibition of JAK2/STAT3 and WNT/β‐catenin signalling pathways

**DOI:** 10.1111/jcmm.15226

**Published:** 2020-04-13

**Authors:** Hai Huang, Ying Han, Zhijun Chen, Xin Pan, Putao Yuan, Xiangde Zhao, Hongfang Zhu, Jiying Wang, Xuewu Sun, Peihua Shi

**Affiliations:** ^1^ Department of Orthopaedic Surgery Sir Run Run Shaw Hospital Zhejiang University School of Medicine Hangzhou China; ^2^ Key Laboratory of Musculoskeletal System Degeneration and Regeneration Translational Research of Zhejiang Province Hangzhou China

**Keywords:** epithelial‐mesenchymal transition, Krüppel‐like factor 5, ML264, osteosarcoma

## Abstract

Osteosarcoma, the most common bone malignancy, has a high morbidity rate and poor prognosis. Krüppel‐like factor 5 (KLF5) is a key transcriptional regulator of cellular proliferation whose overexpression is observed in osteosarcoma cell lines (U2OS, 143B, MG63 and SAOS2). ML264, a small‐molecule inhibitor of KLF5, exerts antiproliferative effects in colorectal cancer; however, its function in osteosarcoma remains unknown. Here, we explored the possible antitumour effects of ML264 on 143B and U2OS cell lines and murine tumour xenograft model. ML264 suppressed proliferation and clonogenic ability of osteosarcoma cells in a dose‐dependent manner. Moreover, ML264 induced G0/G1 cell cycle arrest, with no influence on apoptosis, and inhibited the migratory and invasive abilities of osteosarcoma cells, as demonstrated by wound‐healing and Transwell assays. Exposure to ML264 reduced the mRNA and protein levels of molecules associated with epithelial‐mesenchymal transition phenotype, including N‐cadherin, vimentin, Snail, matrix metalloproteinase (MMP) 9 and MMP13. Inhibition of signal transducer and activator of transcription (STAT) 3 phosphorylation and Wnt signalling was also observed. In the murine model of osteosarcoma, tumour growth was efficiently suppressed following a 10‐day treatment with ML264. Collectively, our findings demonstrate the potential value of ML264 as a novel anticancer drug for osteosarcoma.

## 1 INTRODUCTION

Osteosarcoma is a prevalent, highly malignant bone tumour with high metastatic potential that occurs primarily in children and adolescents.^1^, ^2^ Although the 5‐year survival rate has been greatly increased by a combination of surgery and multi‐drug chemotherapy, osteosarcoma is still associated with a mortality rate of 30%.^3^, ^4^, ^5^ In addition, the survival rates have not improved for nearly two decades suggesting that treatment of osteosarcoma has reached a bottleneck.^6^, ^7^ Patients with recurrent and metastatic osteosarcoma have particularly poor outcome, owing to inadequate response to current conventional chemotherapeutic agents.^8^, ^9^ Therefore, there is an urgent need to identify new, more effective treatment strategies for patients with this lethal disease.

Krüppel‐like factor 5 (KLF5) is a zinc‐finger transcription factor involved in numerous functions in eukaryotic cells, such as proliferation, migration and differentiation.^10^, ^11^ Its overexpression is observed in many human diseases, including intestinal, colon, breast, bladder and pancreatic cancer.^12^, ^13^, ^14^ KLF5 regulates epithelial‐mesenchymal transition (EMT),^15^ which is characterized by loss of cell adhesion and polarity, and acquisition of a mesenchymal‐like phenotype by epithelial cells.^15^, ^16^ EMT is closely related to tumour migration and invasion ^17^, ^18^; however, the underlying molecular mechanisms are yet to be elucidated.

ML264 is a small‐molecule compound that inhibits the expression of KLF5 and its transcriptional activator, early growth response gene 1 (EGR1).^19^ The drug potently suppresses proliferation of colon cancer cells and growth of xenograft tumours in nude mice.^19^ Moreover, a novel derivative of ML264, YD277, inhibits proliferation of triple‐negative breast cancer (TNBC) cells and growth of xenografts by inducing apoptosis and G1 cell cycle arrest.^20^ Although KLF5 activity is implicated in many types of cancer,^12^, ^13^, ^14^ the effects of its inhibition have not been investigated in detail in osteosarcoma. This study explored the effects of ML264 on the viability, growth, metastasis and apoptosis of osteosarcoma cells to evaluate its role in osteosarcoma therapy. The molecular mechanisms and signalling pathways mediating the antitumour effects of ML264 were also investigated. Our study demonstrates that ML264 is a promising therapeutic drug to inhibit the development of osteosarcoma.

## 2 MATERIALS AND METHODS

### 2.1 Reagents and antibodies

The compound ML264 was obtained from The Scripps Research Institute in the laboratory of Dr Thomas Bannister,^21^ and its structure and synthesis pathway have been previously published.^21^ Dimethyl sulphoxide (DMSO) was purchased from Sigma. MEM medium, foetal bovine serum (FBS) and phosphate‐buffered saline (PBS) were obtained from Gibco Life Technologies. Cell counting kit‐8 (CCK‐8) was purchased from Dojindo Molecular Technology. Specific antibodies against Runx2, β‐catenin, p‐β‐catenin, STAT3, p‐STAT3, JAK2, p‐JAK2, JNK, p‐JNK, ERK1/2, p‐ERK1/2, p38, p‐p38, β‐actin and β‐tubulin were obtained from Cell Signaling Technology, and antibodies against EGR1, KLF5, cyclin D1, cyclin E1, CDK4, N‐cadherin, E‐cadherin, vimentin, Snail, MMP‐9 and MMP‐13 were purchased from Abcam.

### 2.2 Cell culture

From the American Type Culture Collection (ATCC), 143B (ATCC: CRL‐8303) and U2OS (ATCC: HTB‐96TM) human osteosarcoma cell lines were purchased. 143B and U2OS cells were maintained in Dulbecco's modified Eagle's medium and RPMI1640 medium supplemented with 1% penicillin/streptomycin and 10% FBS, respectively. These cells were cultured in an incubator at a constant temperature of 37°C with 5% CO2.

### 2.3 CCK‐8 assay

The effect of ML264 on the cell viability of 143B and U2OS cells was examined by CCK‐8 assay. Firstly, 96‐well plates were used for inoculation of 143B and U2OS cells at a density of 4 × 10^3^ cells/well. Then, cells were treated using different concentrations of ML264 (0‐40 μmol/L) for 48 or 96 hours, whereas the negative control was treated using DMSO. At the end of culture, each well was added 10 μL of CCK‐8 buffer and incubated for another 2 hours, after which the absorbance of each well at 450 nm wavelength was measured using an ELX800 absorbance microplate reader (BioTek Instruments).

### 2.4 Colony‐formation assay

Six‐well plates were used for inoculation of 143B and U2OS cells at a density of 1000 cells/well. After adherence for 24 hours, the cells were exposed to 0.5, 1 and 2 μmol/L ML264 and the control medium for 7‐14 days. When the colonies became visible, 4% paraformaldehyde was used for fixation of cells, and 0.5% crystal violet was used for staining. After washing with PBS, the number of colonies was then counted and analysed.

### 2.5 Flow cytometry

Six‐well plates were used for inoculation of 143B and U2OS cells at a density of 5 × 10^5^ cells/well. After adherence for 24 hours, the cells were exposed to different concentrations of ML264 and the control medium for 24 hours. For cell cycle assay, the cells were digested, centrifuged and washed twice. Then, cold 75% ethanol was used for fixation of the cells for at least 2 hours. After washed twice with PBS to eliminate remanent ethanol, the cells were then incubated with RNase A (100 μg/mL), propidium iodide (PI; 50 μg/mL) and 0.2% Triton X‐100 complexes for 15 minutes in the dark. At the end, the distribution of cells in different cell cycles was examined using the Accuri C6 (BD Biosciences). As for apoptosis analysis, the adherent cells were harvested, as well as the cells in medium. After washed twice with PBS, the cells were incubated with FITC‐conjugated Annexin V and PI in the dark for 15 minutes. Then, the samples were analysed using the Accuri C6, too.

### 2.6 Wound‐healing assay

Six‐well plates were used for inoculation of 143B and U2OS cells at a density of 1 × 10^5 ^cells/well. Three perpendicular wounds were scratched using a 200‐μL pipette tip when the cells were grown to about 80% confluency, after which the wells were washed twice with PBS to eliminate the detached cells. Then, the cells were exposed to 0.5, 1 and 2 μmol/L ML264 and the control medium for 24 hours. An inverted microscope (Nikon) was used to photograph the wounds at 0 and 24 hours.

### 2.7 Cell migration and invasion assays

Transwell cell culture chambers were used to assess cell migration and invasion ability of 143B and U2OS cells. Whereas invasion assay needed Matrigel basement membrane matrix (BD Biosciences) coated on the membranes of upper chamber at 37°C for 6 hours, migration assay did not. 143B and U2OS cells were exposed to different concentrations of ML264 for 24 hours, after which 100 μL of cells (5 × 10^4^) were inoculated into the upper chamber in serum‐free media, whereas the lower chamber was added with 600 μL of medium supplemented with 10% FBS. After cultured in incubator for 24 hours, cells which invaded the membrane were fixed and stained using 0.5% crystal violet for 15 minutes. After washed by PBS for five times, the other cells in the upper surface were wiped off carefully. Then, five representative microscopic fields of the migrant cells were imaged and counted (×100 magnification).

### 2.8 Immunofluorescence assay

Forty‐eight‐well plates were used for inoculation of 143B and U2OS cells. After exposed to 2 μmol/L ML264 and DMSO for 24 hours, cells were fixed for 20 minutes and permeabilized using 0.5% Triton X‐100 for another 30 minutes. Whereafter, 5% sheep serum was used for blocking before cells were incubated with primary antibodies of EMT‐related proteins at 4°C overnight. Cells were washed for three times the next day, after which Alexa Fluor 488 secondary antibody was used for incubation at room temperature for 1 hour. At the end, DAPI buffer was used to stain cell nuclei, after which cells were photographed using a fluorescence microscope.

### 2.9 Quantitative real‐time PCR

RT‐PCR was carried out to assess KLF5, EGR1, cell cycle‐associated genes and EMT‐associated genes expression levels in osteosarcoma cells after ML264 treatment. Six‐well plates were used for inoculation of 143B and U2OS cells at a density of 5 × 10^4^ cells/well. After adherence for 24 hours, the cells were exposed to different concentrations of ML264 (0.5, 1 or 2 μmol/L) for 48 hours or 2 μmol/L of ML264 for different durations (6, 12, 24 hours). According to the manufacturer's instruction, total RNA of the cultured cells was extracted using RNeasy kit (Invitrogen), which was used to synthesize complementary DNA (cDNA) with the 5 × PrimeScript RT Master Mix (Takara Bio) for mRNA analysis. An ABI Prism 7500 system (Applied Biosystems) was subsequently used for RT‐PCR with SYBR Green QPCR Master Mix (Takara Bio). The transcriptional levels of target gene were calculated using the 2^‐ΔΔCt^ method, and the concentrations of cDNA in each sample were verified by β‐actin. The specific primers used in this study were listed in Table 1.

**Table 1:** The sequences of primers used in this study

Gene	Primers
β‐Actin	Forward: 5′‐GATGAGATTGGCATGGCTTT‐3′
	Reverse: 5′‐CACCTTCACCGTTCCAGTTT‐3′
cyclin D1	Forward: 5′‐GCTGCGAAGTGGAAACCATC‐3′
	Reverse: 5′‐CCTCCTTCTGCACACATTTGAA‐3′
cyclin E1	Forward: 5′‐GCCAGCCTTGGGACAATAATG‐3′
	Reverse: 5′‐CTTGCACGTTGAGTTTGGGT‐3′
CDK4	Forward: 5′‐ATGGCTACCTCTCGATATGAGC‐3′
	Reverse: 5′‐CATTGGGGACTCTCACACTCT‐3′
E‐cadherin	Forward: 5′‐CTCGACACCCGATTCAAAGT‐3′
	Reverse: 5′‐GCGTGACTTTGGTGGAAAAC‐3′
N‐cadherin	Forward: 5′‐AGGATCAACCCCATACACCA‐3′
	Reverse: 5′‐TGGTTTGACCACGGTGACTA‐3′
Vimentin	Forward: 5′‐AGTCCACTGAGTACCGGAGAC‐3′
	Reverse: 5′‐CATTTCACGCATCTGGCGTTC‐3′
Snail	Forward: 5′‐TTTACCTTCCAGCAGCCCTA‐3′
	Reverse: 5′‐GGACAGAGTCCCAGATGAGC‐3′
MMP9	Forward: 5′‐TGTACCGCTATGGTTACACTCG‐3′
	Reverse : 5′‐GGCAGGGACAGTTGCTTCT‐3′
MMP13	Forward: 5′‐ACTGAGAGGCTCCGAGAAATG‐3′
	Reverse : 5′‐GAACCCCGCATCTTGGCTT‐3′
EGR1	Forward: 5′‐GGTCAGTGGCCTAGTGAGC‐3′
	Reverse : 5′‐GTGCCGCTGAGTAAATGGGA‐3′
KLF5	Forward: 5′‐CCTGGTCCAGACAAGATGTGA‐3′
	Reverse: 5′‐GAACTGGTCTACGACTGAGGC‐3′
RUNX2	Forward: 5′‐TCAACGATCTGAGATTTGTGGG‐3′
	Reverse: 5′‐GGGGAGGATTTGTGAAGACGG‐3′

### 2.10 Western blotting analysis

Six‐well plates were used for inoculation of 143B and U2OS cells at a density of 5 × 10^4 ^cells/well. Then, cells were exposed to different treatments, after which total proteins were extracted using radioimmunoprecipitation assay (RIPA) lysis buffer (Sigma‐Aldrich) containing a protease and a phosphatase inhibitor. Using SDS‐PAGE (8%‐12%), total proteins were separated and transferred to 0.45‐μm PVDF membranes (Bio‐Rad). Whereafter, 5% non‐fat dry milk was used for blocking of the membranes at room temperature for 1 hour, after which specific primary antibodies were used for incubation overnight at 4°C. After these membranes were washed with TBST buffer for three times, corresponding secondary antibody was used for incubation at room temperature for 1 hour. And then, a Chemiluminescence Kit (Millipore) was used to detect the protein band. The protein levels were first normalized to β‐actin or α‐tubulin, and then normalized to experimental controls.

### 2.11 OS mouse model

For our in vivo tumour experiments, 4‐week‐old female BALB/c‐nu mice (Shanghai SLAC Laboratory Animal Co., Ltd.) were purchased. 143B cells were digested, centrifuged and suspended in PBS at a concentration of 1 × 10^7^/mL. One hundred micro litre cell suspension was injected subcutaneously into the flank of each nude mice. After 10 days, these mice were separated into three groups at random and given injection of DMSO and ML264 (20 and 40 mg/kg/d) intraperitoneally. Tumour volume and mice weight were measured every day, using a digital caliper and a weigher. After ten times of drug administration, the mice were all killed. The tumours were removed from the flank of each nude mice for further analysis, and their weight was measured. All the animal‐related experiments were approved by the Animal Care and Use Committee of Sir Run Run Shaw Hospital.

### 2.12 Histopathology and immunohistochemistry

The viscera, such as heart, liver, spleen, lung and kidney, were gutted from three groups of mice and fixed with formalin, after which they were embedded with paraffin. Then, these cut slices were stained with haematoxylin and eosin (H&E) before they were photographed for further histological examinations. As for tumour tissue, they were embedded for immunohistochemical analysis as described previously.^22^

### 2.13 Statistical analysis

All quantitative data are presented as mean ± SEM. Statistical significance was analysed with the unpaired, two‐tailed Student's *t* test or ANOVA for multiple comparisons. The value of *P* < .05 was considered to be statistically significant. Significance level was presented as either *P < .05, **P < .01 or ***P < .001.

## 3 RESULTS

### 3.1 ML264 inhibits proliferation of 143B and U2OS cells without inducing apoptosis

**FIGURE 1:**
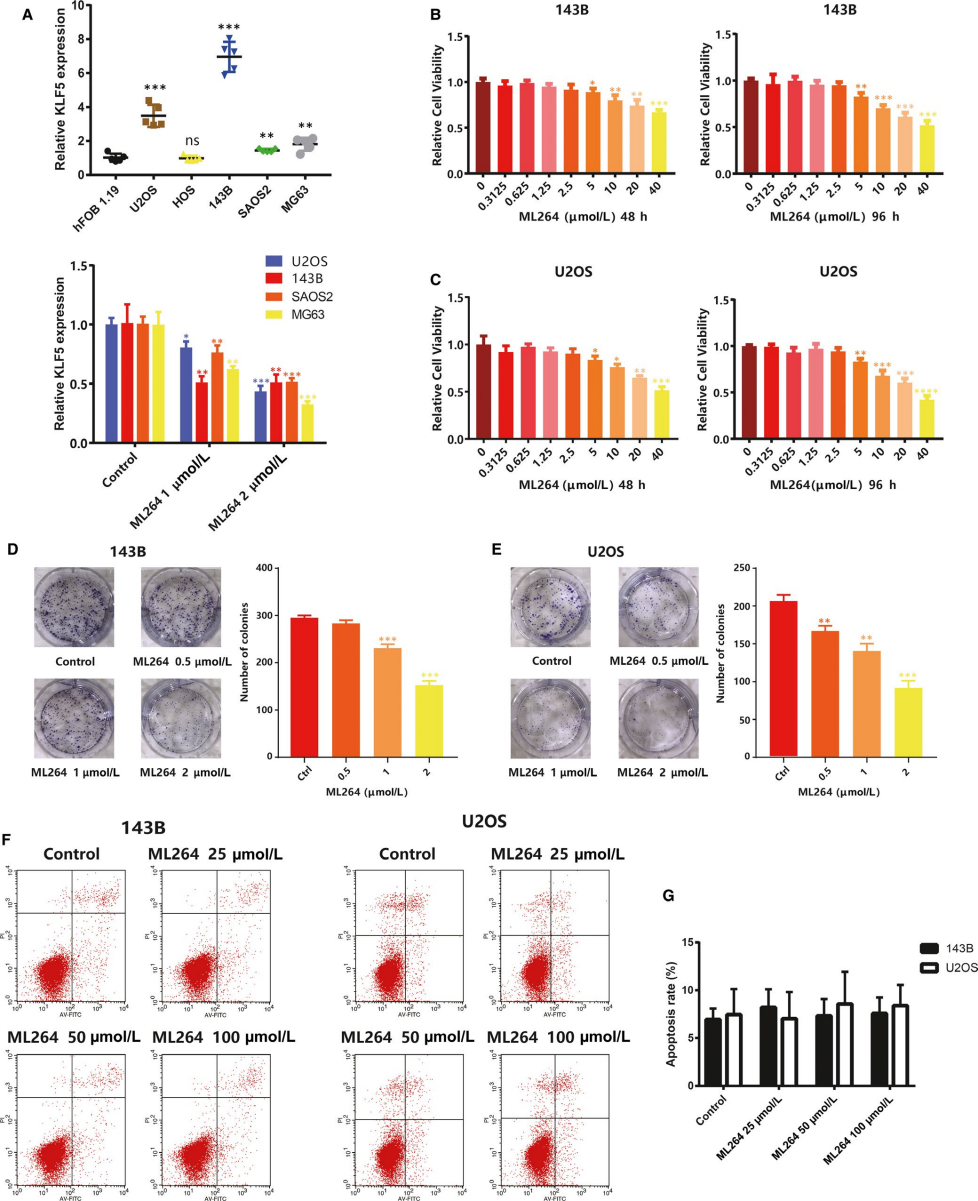
ML264 inhibits proliferation of 143B and U2OS cells without inducing apoptosis. A, The mRNA expression of KLF5 in hFOB1.19 and osteosarcoma cell lines with or without ML264 exposure. B and C, The antiproliferative effect of ML264 on osteosarcoma cell lines was determined by CCK‐8 assays. 143B and U2OS cells were treated with different concentrations of ML264 for 48 and 96 h. Control group contained 0.1% DMSO. D and E, Colony‐formation assay of 143B and U2OS cells with 0.1% DMSO or ML264. F, 143B and U2OS cells were treated with different concentrations of ML264 for 48 h, stained with PI and V‐FITC, and analysed by flow cytometry to determine the percentage of cells in apoptosis. G, The percentage of cells in apoptosis was quantified for each treatment. Data are expressed as the means ± SD of three independent experiments. *P < .05, **P < .01, ***P < .001 compared with the control group. CCK‐8, cell counting kit‐8; DMSO, dimethyl sulphoxide; FITC: fluorescein isothiocyanate; PI, propidium iodide

First, we measured KLF5 expression in hFOB1.19 cells (human osteoblast cell line) and different osteosarcoma cell lines using qRT‐PCR. The results showed that KLF5 expression was up‐regulated in osteosarcoma, most significantly in 143B and U2OS cells. Following ML264 exposure, KLF5 expression was significantly down‐regulated (Figure 1A). Subsequently, 143B and U2OS cells were treated with different concentrations of ML264 (0‐40 μmol/L) for 48 or 96 hours, after which cell viability was tested using the Cell Counting Kit‐8 (CCK‐8) (Figure 1B,C). The IC50 value for ML264 was 62 μmol/L at 48 hours and 32.75 μmol/L at 96 hours in 143B cells and 35.77 μmol/L at 48 hours and 27.3 μmol/L at 96 hours in U2OS cells. Additionally, the colony‐forming ability of the cells was weaker following treatment with ML264 (Figure 1D,E and Figure S1a,b). However, exposure to ML264 (25, 50 and 100 μmol/L) did not influence apoptosis rates in 143B or U2OS cells (Figure 1F,G). The above results show that ML264 inhibits the growth of osteosarcoma cells in a dose‐ and time‐dependent manner without inducing apoptosis.

### 3.2 ML264 induces G0/G1 phase arrest by regulating cell cycle‐related proteins

**FIGURE 2:**
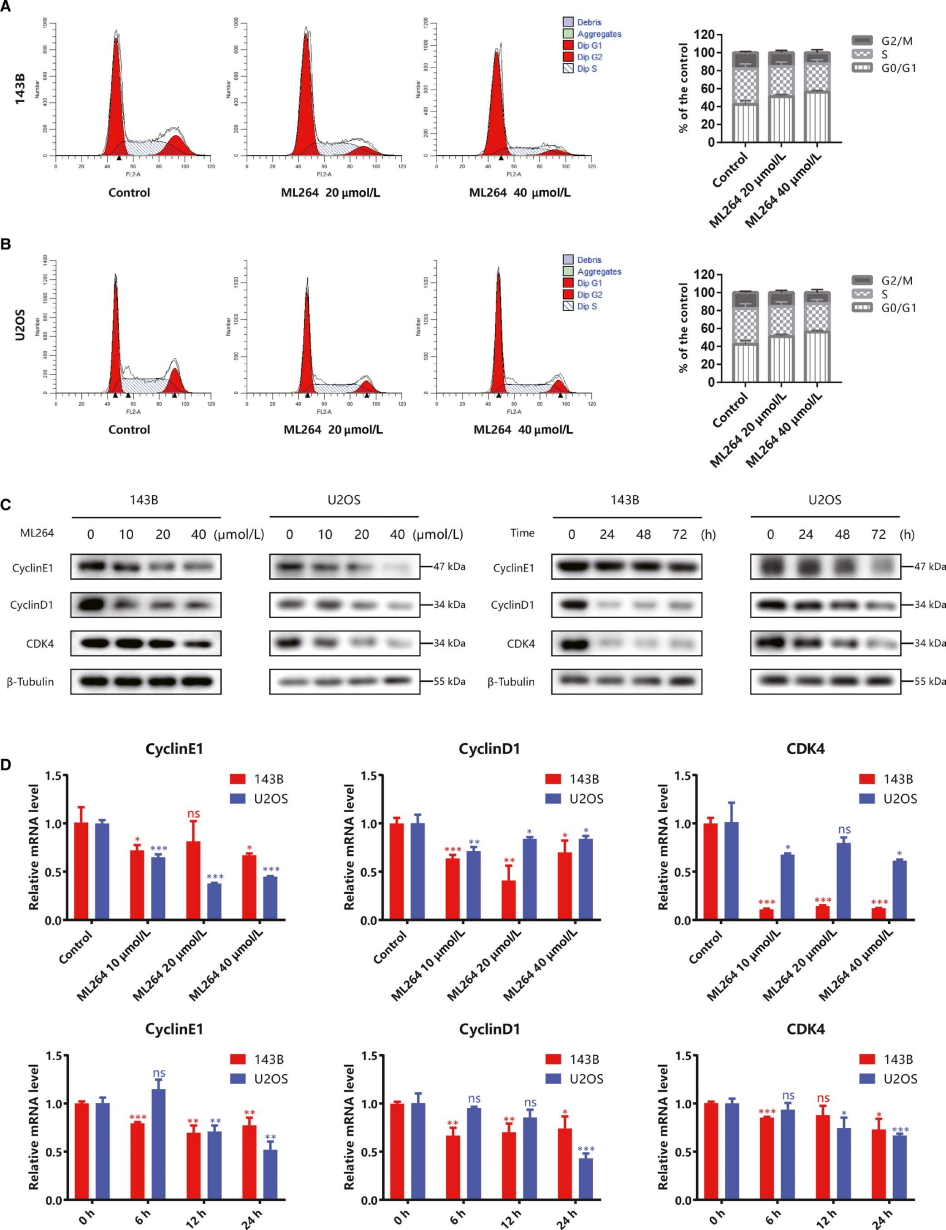
ML264 induces G0/G1 phase arrest by regulating cell cycle‐related proteins. A and B, 143B and U2OS cells were treated with 0.1% DMSO or ML264 for 24 h, and cell cycle assays were performed with flow cytometry. C, 143B and U2OS cells were treated with different doses of ML264 (10, 20 or 40 μmol/L) for 48 h, or 20 μmol/L of ML264 for different durations (24, 48 or 72 h). The expressions of cell cycle‐regulated proteins were then measured using Western blotting. D, The grey levels of cell cycle‐regulated proteins were quantified and normalized to that of β‐tubulin using ImageJ. Data are expressed as the means ± SD of three independent experiments. *P < .05, **P < .01, ***P < .001 compared with the control group. DMSO, dimethyl sulphoxide; PI, propidium iodide

As ML264 inhibited cell proliferation without an effect on apoptosis, we investigated its ability to induce cell cycle arrest. We assessed the distribution of 143B and U2OS cells in the different cell cycle phases following a 24‐hours incubation with ML264 (20 or 40 μmol/L). ML264 treatment increased the proportion of G0/G1 phase cells while decreasing the number of G2/M and S phase cells (Figure 2A,B). To delineate the mechanisms mediating ML264‐induced cell cycle arrest, we examined the expression of cell cycle‐related proteins. ML264 decreased the expression levels of cyclin E1, cyclin D1 and cyclin‐dependent kinase (CDK) 4 in both a dose‐ and time‐dependent manner (Figure 2C), and mRNA expressions were approximately similar to the proteins (Figure 2D). The above results indicate that ML264 therapy could down‐regulate the expression of proteins associated with G0/G1 cell cycle regulation in osteosarcoma cells, thereby inducing G0/G1 phase arrest.

### 3.3 ML264 inhibits invasion and migration in 143B and U2OS cells

**FIGURE 3:**
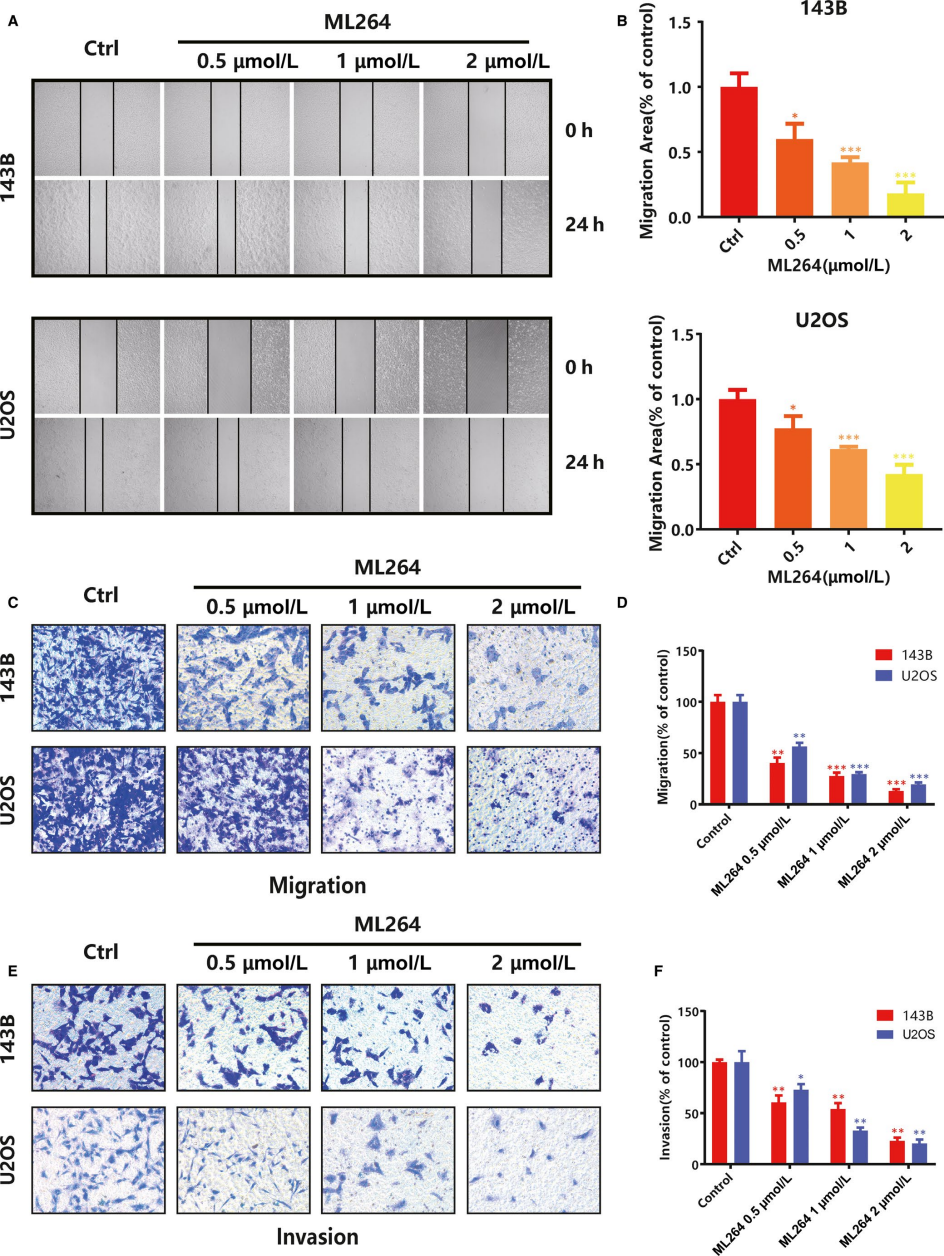
ML264 inhibits invasion and migration in 143B and U2OS cells. A, 143B and U2OS cells were scratched using 200‐μL pipette tips, after which they were treated with different concentrations of ML264 for 24 h. B, The migration area was qualified. C, Cell migration assay was performed using a Transwell chambers for 24 h. D, The migration abilities of 143B and U2OS cells were quantified. E, Cell invasion assay was performed using Matrigel‐coated Transwell chambers for 24 h. F, The invasion abilities of 143B and U2OS cells were quantified. Data are expressed as the means ± SD of three independent experiments. *P < .05, **P < .01, ***P < .001 compared with the control group

We conducted wound‐healing and Transwell assays to assess the effects of ML264 on the migratory and invasive abilities of osteosarcoma cells. Treatment with ML264 (0.5, 1 and 2 μmol/L) for 24 hours reduced cell migration into scratch wounds in a dose‐dependent manner in both 143B and U2OS cells (Figure 3A,B and Figure S1c,d). Similarly, in the Transwell assays, ML264 decreased the relative migration rates of osteosarcoma cells in a dose‐dependent manner (Figure 3C,D), as well as the invasion rates (Figure 3E,F). The above results suggest that ML264 treatment is able to suppress the metastatic capacity of osteosarcoma cells.

### 3.4 ML264 reverses epithelial‐mesenchymal transition (EMT) phenotype in vitro

**FIGURE 4:**
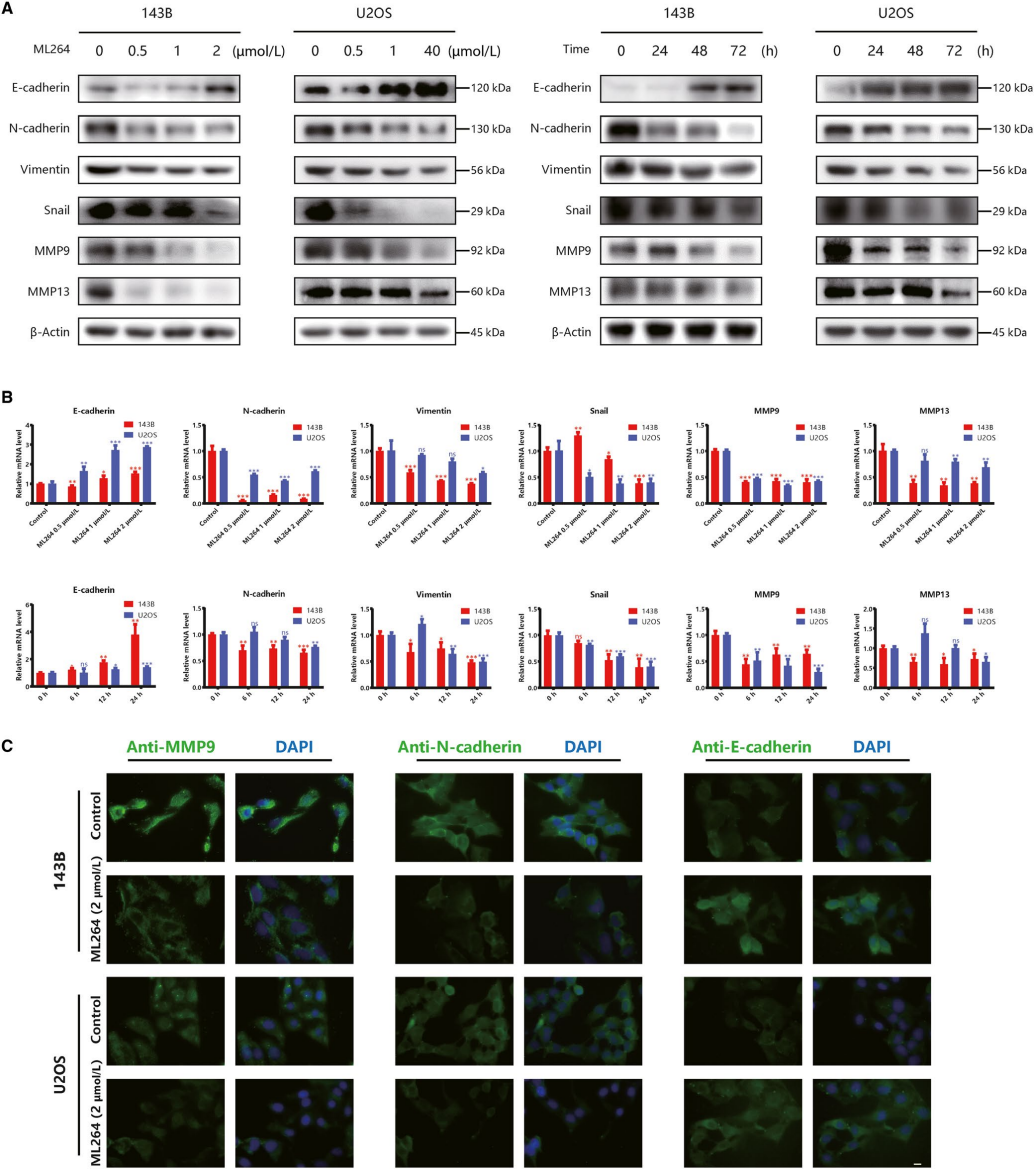
ML264 reverses epithelial‐mesenchymal transition (EMT) phenotype in vitro. A, 143B and U2OS cells were treated with different doses of ML264 (0.5, 1 or 2 μmol/L) for 48 h, or 2 μmol/L of ML264 for different durations (24, 48 or 72 h). The expressions of EMT‐regulated proteins were measured by Western blotting. B, The expressions of EMT‐regulated mRNA were measured by RT‐PCR. C, The level of MMP9, N‐cadherin and E‐cadherin in 143B and U2OS cells are presented by immunofluorescence. Data are expressed as the means ± SD of three independent experiments. *P < .05, **P < .01, ***P < .001 compared with the control group. EMT: epithelial‐mesenchymal transition; RT‐PCR: reverse transcription polymerase chain reaction

EMT is thought to be closely associated with tumour progression and metastasis^23^; thus, we investigated whether ML264 inhibits cell migration by reversing the EMT phenotype. We measured the expression of EMT‐related proteins in ML264‐treated osteosarcoma cells (0.5, 1 or 2 μmol/L) and found that exposure to ML264 significantly down‐regulated the mRNA and protein levels of mesenchymal markers (N‐cadherin, vimentin, Snail, MMP9 and MMP13) and up‐regulated the levels of epithelial cell marker E‐cadherin, in a time‐ and dose‐dependent manner (Figure 4A,B). In line with these results, the immunofluorescence assay indicated decreased MMP9 and N‐cadherin expression and increased E‐cadherin levels following ML264 treatment (Figure 4C). Hence, ML264 reversed the EMT phenotype to inhibit metastatic progression in osteosarcoma cells.

### 3.5 ML264 inhibits the expression of EGR1 and KLF5

**FIGURE 5:**
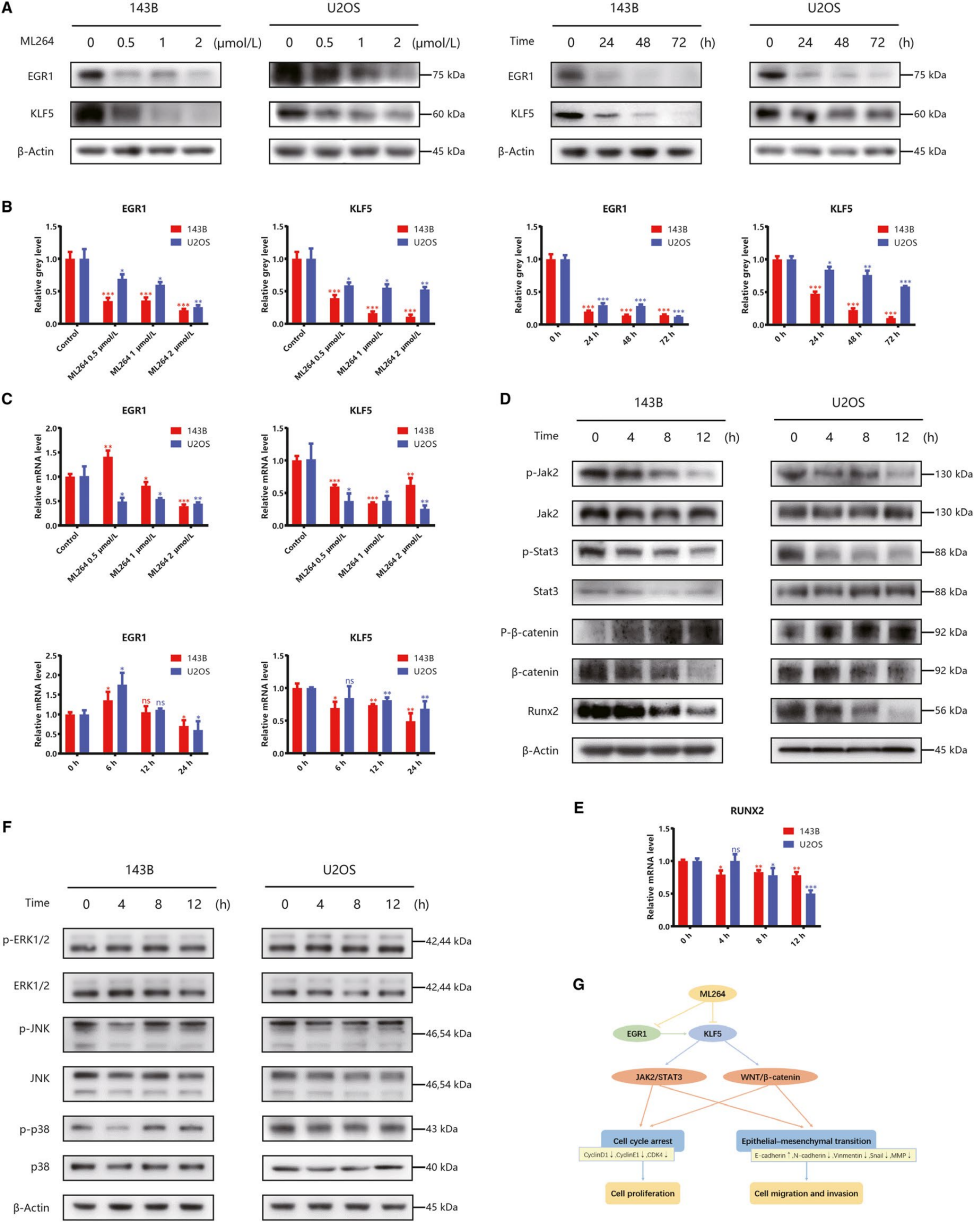
ML264 inhibits the expression of EGR1, KLF5 and JAK2/STAT3 and Wnt/β‐catenin signalling. A, 143B and U2OS cells were treated with different doses of ML264 (0.5, 1 or 2 μmol/L) for 48 h, or 2 μmol/L of ML264 for different durations (24, 48 or 72 h). The protein expressions of EGR1 and KLF5 were measured using Western blotting. B, The grey levels of EGR1 and KLF5 were quantified and normalized to that of β‐actin using ImageJ. C, The mRNA expressions of EGR1 and KLF5 were measured by RT‐PCR. D‐F, The expressions of JAK2/STAT3, WNT and MAPK pathway‐related proteins were measured using Western blotting and RT‐PCR. G, Schematic diagram of the mechanism by which ML264 works. Data are expressed as the means ± SD of three independent experiments. *P < .05, **P < .01, ***P < .001 compared with the control group. RT‐PCR: reverse transcription polymerase chain reaction

ML264 was originally synthesized as an inhibitor of KLF5; thus, we examined the expression of KLF5 and its transcriptional activator, EGR1, in 143B and U2OS cells following ML264 exposure. As expected, ML264 treatment reduced the mRNA and protein levels of both EGR1 and KLF5 in a dose‐ and time‐dependent manner (Figure 5). Thus, ML264 may exert its effects by altering the expression of EGR1, KLF5 and their downstream proteins.

### 3.6 ML264 inhibits JAK2/STAT3 and Wnt/β‐catenin signalling

As KLF5 is a positive regulator of both the JAK2/STAT3 and Wnt/β‐catenin signalling pathways,^24^, ^25^, ^26^, ^27^ we used Western blotting to measure the expression of related proteins in ML264‐treated cells. ML264 exposure decreased the phosphorylation of JAK2 and STAT3 compared with control levels. Simultaneously, it down‐regulated protein levels of Runx2 and total β‐catenin in a time‐dependent manner and increased the levels of phospho‐β‐catenin (Thr41/Ser45), which impaired Wnt signalling (Figure 5D). In line with these results, ML264 inhibited mRNA expression of Runx2 in a time‐dependent manner (Figure 5E). Notably, MAPK pathway‐related proteins were not influenced by ML264 treatment (Figure 5F). In conclusion, ML264 inhibited the JAK2/STAT3 and Wnt/β‐catenin signalling pathways via down‐regulation of KLF5 and EGR1 expression, thereby inhibiting proliferation and metastasis of osteosarcoma cells (Figure 5G).

### 3.7 ML264 inhibits the growth of osteosarcoma in vivo

**FIGURE 6:**
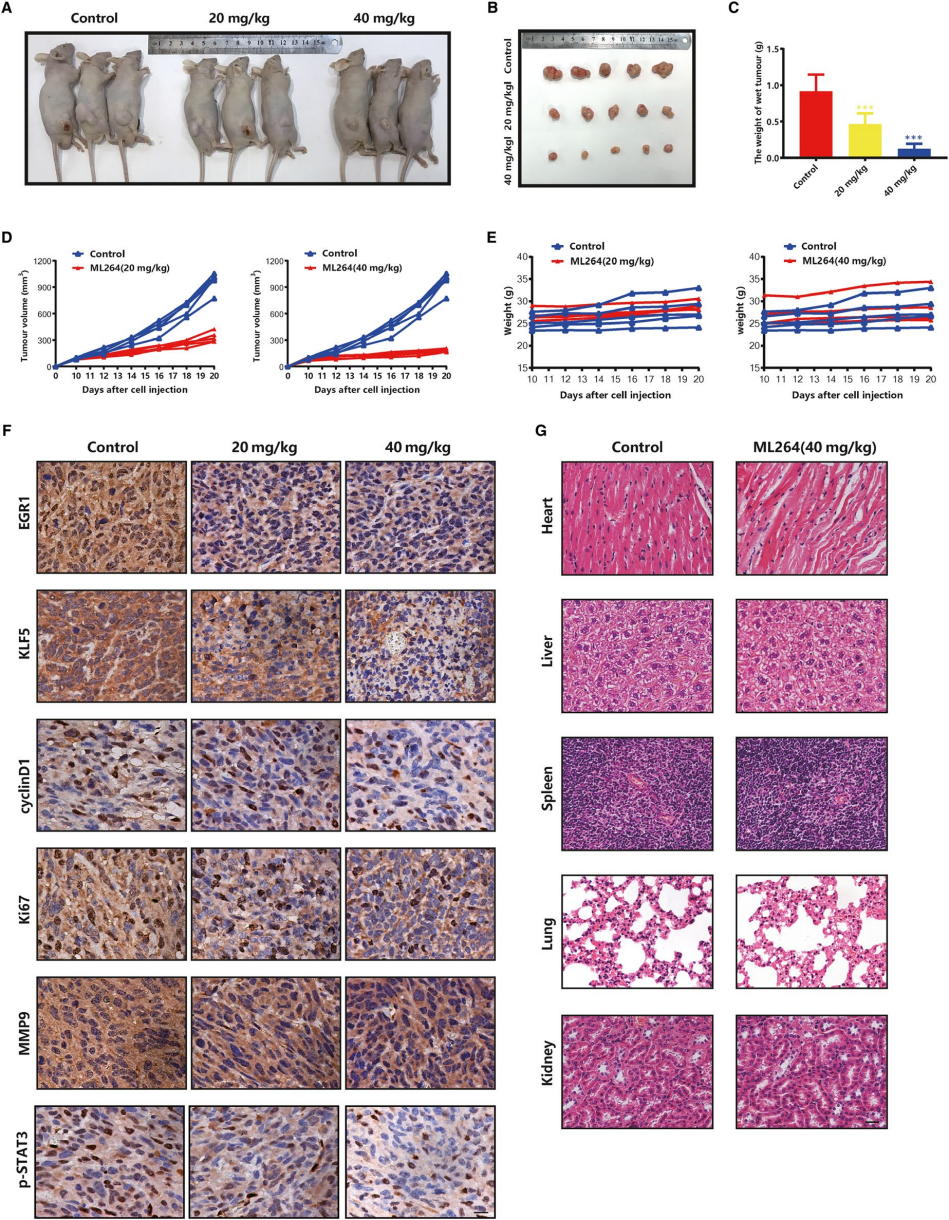
ML264 inhibits the growth of osteosarcoma in vivo. 143B cells were injected into the flanks of nude mice. After 10 d, mice were randomly divided into three groups and administered intraperitoneal injection of DMSO and ML264 (20 and 40 mg/kg/d), for 10 days, respectively. A, Tumour status was presented. B, Tumours were separated from killed mice. C, The tumours were weighed and analysed. D and E, The tumour volume and weight of mice were measured daily. F, Representative pictures of EGR1, KLF5, cyclinD1, Ki67, MMP9 and p‐STAT3 immunostaining. G, H&E staining of major organs is presented. Data are expressed as the means ± SD of three independent experiments. *P < .05, **P < .01, ***P < .001 compared with the control group. DMSO: dimethyl sulphoxide

We next sought to evaluate the effects of ML264 on osteosarcoma in vivo. A tumour xenograft model was established via injection of 143B cells into the flanks of nude mice. After 10 days, the mice were randomized into three groups and intraperitoneally administered DMSO (control) or ML264 (20 or 40 mg/kg bodyweight/day) for 10 days. ML264 suppressed the growth of xenograft tumours in a dose‐dependent manner (Figure 6A‐D) without significantly affecting the bodyweight of the animals (Figure 6E). In addition, immunohistochemical analyses indicated that exposure to ML264 decreased the expression levels of EGR1, KLF5, cyclin D1, Ki67, MMP9 and phospho‐STAT3 (Figure 6F). To investigate whether ML264 is cytotoxic to non‐tumour cells, we performed H&E staining on tissue harvested from DMSO‐ and ML264‐treated mice. No toxic effects were found in the major organs (Figure 6G). Taken together, these results suggest that ML264 hinders the development of osteosarcoma and has low toxicity in nude mice in vivo.

## 4 DISCUSSION

Osteosarcoma is a highly malignant tumour with high metastatic potential and poor survival rate despite multimodal treatment. The unfavourable prognosis of the disease necessitates the development of novel, more effective treatment approaches to improve patient outcome. ML264 reportedly hinders proliferation of colon cancer cells and growth of xenograft tumours in nude mice.^19^ A derivative of ML264, YD277, has demonstrated similar effects in TNBC cells.^20^ The current study explored the inhibitory effects of ML264 on osteosarcoma cells. In brief, ML264 inhibited the JAK2/STAT3 and Wnt/β‐catenin signalling pathways by decreasing the levels of KLF5 and EGR1, and down‐regulated the expression of proteins associated with cell cycle regulation and EMT.

KLF5 is one of the basic transcriptional factors, ubiquitously expressed in different tissues.^28^ It is associated with numerous cell functions, including proliferation, differentiation, migration and EMT.^10^, ^11^, ^15^ In our study, ML264 reduced the expression of KLF5 at both the transcriptional and protein level, which, in turn, altered the expression of various downstream molecules to hinder proliferation and metastasis of osteosarcoma cells.

A previous study reported that ML264 affects colorectal cancer proliferation by regulating the RAS/MAPK/PI3K and Wnt pathways.^19^ In our study, the antitumour effects of ML264 on 143B and U2OS cell lines were mediated via inhibition of JAK2/STAT3 and Wnt/β‐catenin signalling. Mechanistically, KLF5 is a positive regulator of the JAK2/STAT3 and WNnt/β‐catenin signalling pathways. Its disruption stimulates the expression of N‐myc downstream‐regulated gene 2 (NDRG2),^24^ a tumour suppressor^25^ that can inhibit STAT3 signalling,^26^ whereas activation of STAT3 is involved in cyclin D1 overexpression.^29^ Moreover, it is accepted that the phosphorylation of STAT3 is involved in EMT and tumour metastasis. To be specific, several molecules have been demonstrated to promote tumour metastasis by activating the STAT3 pathway.^30^, ^31^ Accordingly, suppression of STAT3 phosphorylation down‐regulated the expression of mesenchymal markers (N‐cadherin, Snail and MMP9) and increased the levels of E‐cadherin, an epithelial cell marker.^32^ Additionally, KLF5 can increase the expression of β‐catenin and directly interact with β‐catenin to stabilize it and promote its nuclear transfer.^27^ β‐catenin binds to transcription factors of the T cell factor/ lymphocyte enhancer factor (TCF/LEF) family, leading to the activation of downstream genes, such as cyclin D1.^33^ Furthermore, β‐catenin binds E‐cadherin, and thus, it is a crucial converging element between the EMT and canonical Wnt signalling.^34^, ^35^ During EMT, decreased levels of E‐cadherin lead to the accumulation of β‐catenin and activation of Wnt signalling. Conversely, decreased β‐catenin levels stimulate E‐cadherin accumulation and inhibit the EMT process. In conclusion, ML264 inhibits osteosarcoma growth and metastasis by inhibiting the JAK2/STAT3 and Wnt signalling pathways.

In summary, the above results indicate that ML264 displays potent antitumour effects. The drug hindered proliferation of osteosarcoma cells in vitro and suppressed the growth of xenograft tumours in nude mice. Furthermore, ML264 induced G0/G1 cell cycle arrest and impaired cellular migration, invasion and EMT. The inhibitory effects of ML264 on osteosarcoma cells are likely mediated via inhibition of JAK2 and STAT3 phosphorylation, decrease in β‐catenin and Runx2 levels, down‐regulation of cyclin D1, cyclin E1, CDK4, N‐cadherin, vimentin, Snail and MMPs expression, and up‐regulation of E‐cadherin levels. Therefore, ML264 might be an excellent therapeutic agent to target osteosarcoma as well as other cancer types.

## CONFLICT OF INTEREST

There are no conflicts of interest to declare.

## AUTHOR CONTRIBUTIONS

Peihua Shi, Xuewu Sun and Hai Huang designed this study. Hai Huang, Ying Han and Zhijun Chen conducted the study. Hai Huang, Ying Han and Xin Pan performed in vitro experiments. Hai Huang, Ying Han and Putao Yuan performed in vivo experiments. Zhijun Chen and Xiangde Zhao collected the data and analysed. Hai Huang drafted the manuscript. Hongfang Zhu and Xiangde Zhao revised the manuscript content. J. Wang performed the funding management. P. Shi takes responsibility for the integrity of the data. All authors interpreted the data and approved final version of manuscript.

## Supporting information

Fig S1

## Data Availability

The data used to support the findings of this study are available from the corresponding author upon request.
