# Supplementary figures and images for: ML264 inhibits osteosarcoma growth and metastasis via inhibition of JAK2/STAT3 and WNT/β‐catenin signalling pathways
Source: J Cell Mol Med. 2020 Apr 13;24(10):5652–64. doi: 10.1111/jcmm.15226 (PMC7214147; doi:10.1111/jcmm.15226)

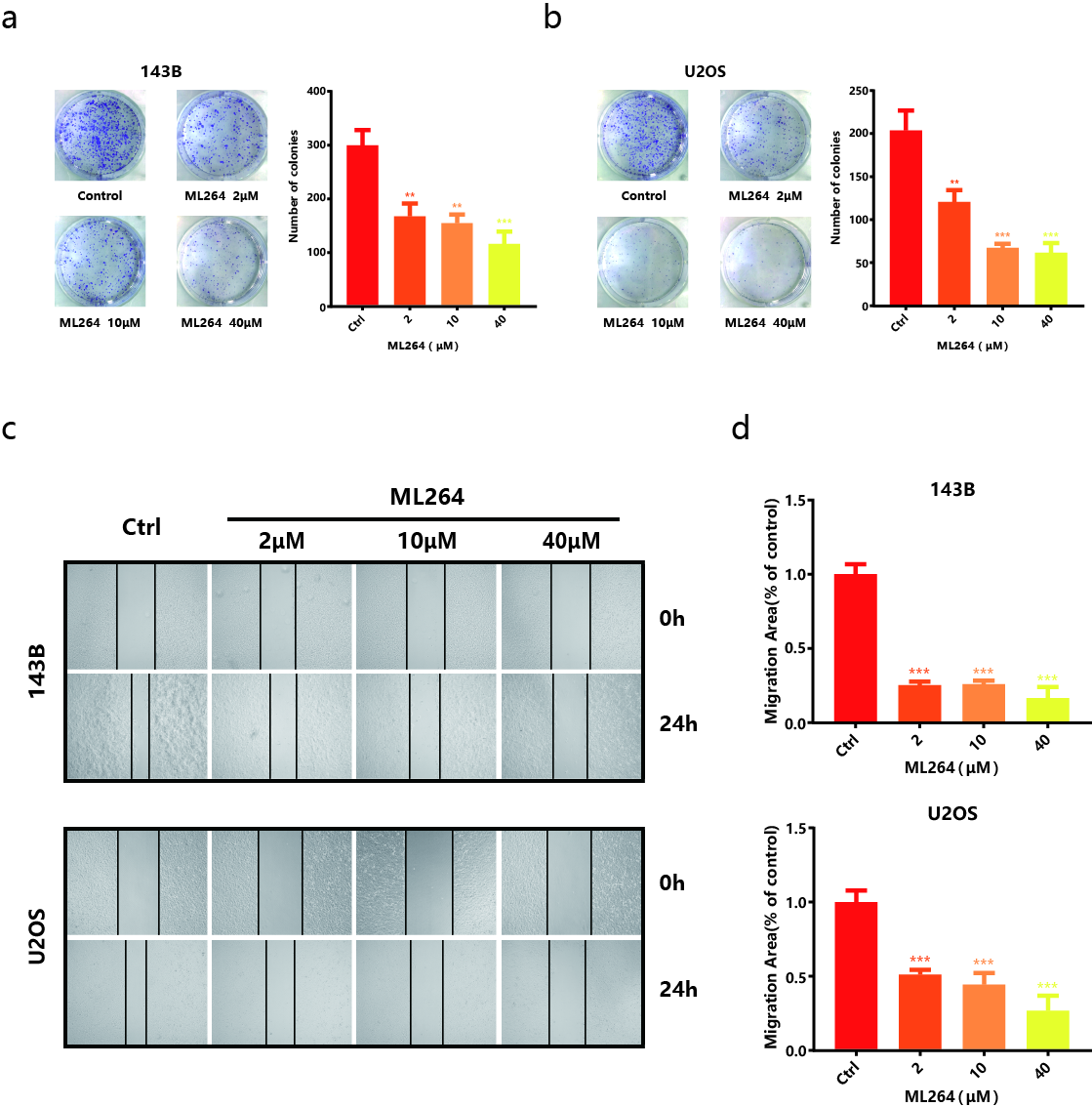

Supplement: Supplementary file 1 — Fig S1 [file JCMM-24-5652-s001.tif]
